# Exploring the Pathways Between Transformative Group Experiences and Identity Fusion

**DOI:** 10.3389/fpsyg.2020.01172

**Published:** 2020-06-03

**Authors:** Christopher M. Kavanagh, Rohan Kapitány, Idhamsyah Eka Putra, Harvey Whitehouse

**Affiliations:** ^1^Centre for the Study of Social Cohesion, University of Oxford, Oxford, United Kingdom; ^2^Institute of Cognitive and Evolutionary Anthropology, University of Oxford, Oxford, United Kingdom; ^3^Department of Psychology, Rikkyo University, Tokyo, Japan; ^4^Faculty of Psychology, Persada Indonesia University, Jakarta, Indonesia; ^5^Division for Applied Social Psychology Research, Jakarta, Indonesia

**Keywords:** social identity, identity fusion, fusion, Islam, Indonesia, extremism

## Abstract

A growing body of evidence suggests that two distinct forms of group alignment are possible: identification and fusion (the former asserts that group and personal identity are distinct, while the latter asserts group and personal identities are functionally equivalent and mutually reinforcing). Among highly fused individuals, group identity taps directly into personal agency and so any attack on the group is perceived as a personal attack and motivates a willingness to fight and possibly even die as a defensive response. As such, identity fusion is relevant in explaining violent extremism, including suicidal terrorist attacks. Identity fusion is theorized to arise as a result from experiences which are (1) perceived as shared and (2) transformative, however evidence for this relationship remains limited. Here, we present a pre-registered study in which we examine the role of transformativeness and perceived sharedness of group-defining events in generating identity fusion. We find that both of these factors are predictive of identity fusion but that the relationship with transformativeness was more consistent than perceived sharedness across analyses in a sample of Indonesian Muslims.

## Introduction

For decades, psychologists have understood group alignment in terms of group identification as outlined by the social identity approach, which combines social identity theory (SIT) and self-categorization theory (Tajfel and Turner, 1985; Haslam et al., 1996; Hogg, 2006; Hornsey, 2008). An important component of this approach is that it posits a “functional antagonism” between social and personal identities (Hogg and Turner, 1987; Turner, 1987). That is, there exists a hydraulic relationship between levels of identity such that making a group identity salient means that one’s personal identity becomes less accessible and vice versa (Tajfel and Turner, 1985). Related to this is the process of *depersonalization* whereby committed group members in salient group contexts perceive themselves less as individuals and more as interchangeable exemplars of the relevant group (Hogg and Turner, 1987; Rosenberg, 1987). Kruglanski et al. (2009, 2014) and Dugas and Kruglanski (2014) work on radicalization emphasizes how such a mechanism plays an important role in ‘quests for significance.’ They describe how individuals undergoing a search for meaning within a group context involves a *collectivist shift* in which there is a “transition from one’s individual identity to one’s social identity as the member of some group” which offers “a sense of empowerment… from identifying with a stronger, more robust and enduring entity whose existence transcends the fragile lives of individual members” (Kruglanski et al., 2019, p. 94).

While there is some evidence for a collectivist shift, with increasing reference to group framing and group goals observed amongst violent terrorists (Kruglanski et al., 2009), it is unclear whether this is driven by processes of depersonalization. Moreover, wider criticisms have been raised concerning the lack of direct evidence for functional antagonism (Sim et al., 2014). Indeed, even advocates of SIT acknowledge that the original model of functional antagonism may be “rigid and over-simplified” (Hornsey, 2008, p. 217). Alternatively, researchers have argued that personal and social identities are fundamentally confounded (Cohen and Garcia, 2005) with some self-affirmation theorists arguing that defense of “both types of identities contribute to the same overarching goal of maintaining self-integrity” (Sherman and Cohen, 2006, p. 206). Relatedly, Swann et al. (2009) proposed an alternative form of group alignment known as ‘identity fusion,’ in which the relationship between personal and group identity^1^ is synergistic rather than hydraulic (Turner, 1987, p. 49): thinking about one’s group identity taps directly into personal agency and vice versa This means that relational ties with group members remain important and capable of motivating actions even in the case of individuals who are highly fused with their group (Buhrmester et al., 2014; Swann et al., 2014).

Drawing on decades of anthropological research on group cohesion in ritual communities, Whitehouse (1992, 2018) and Whitehouse and Lanman (2014) have argued that these divergent forms of group alignment, identification and fusion, are associated with distinctive ritual practices and socioecological contexts. In their proposed model, repeated social interactions in which individuals create semantic memory for group identity markers, including regularly repeated rituals and conventions, produces identification. Since these group identities are acquired from others, via individual or social learning, and are stored as semantic memories they are not attached to distinct episodes of personal life experiences. In contrast, episodic and potentially idiosyncratic memories for events become associated with autobiographical identity and produce identity fusion. It is theorized that when these autobiographical experiences are perceived as being shared with other group members they can produce a fusion of personal and group identities generating the synergistic bonds of identity fusion. One example of the consequences of fusion within an Indonesian context is recounted by Putra and Sukabdi (2013) who describe how members of an Indonesian terrorist group reported personal indignation when their group was attacked or mocked (see also Milla et al., 2019). The key distinction between identity fusion and the related construct of identity integration is that fusion is focused exclusively on the relationship between social groups and an individual’s sense of self not the intra-relationships between different identity domains (Syed and McLean, 2016).

Recently, Whitehouse (2018, p. 2) outlined his ‘shared experiences pathway to fusion’ model, which built on his earlier theoretical work (Whitehouse, 2013) and proposed a “new general theory of extreme self-sacrifice.” The ‘shared pathway’ model presents a causal chain leading to self-sacrifice which begins with the experience of a catalytic emotional event, and a subsequent process of reflection and meaning making, resulting in the sense of possessing a ‘shared essence’ with a group. Recognition of this ‘shared essence’ can be generated either by perceptions of phenotypic similarity (Dar-Nimrod and Heine, 2011; Vázquez et al., 2017), or a sense that episodic memories of self-defining ‘group event’ are shared with other members. Importantly, such a perception need not be accurate. According to the model, the feeling of a shared essence in turn produces identity fusion with the relevant group and this, under conditions of threat, motivates extreme self-sacrifice. Perceptions of having a shared essence are proposed as a necessary condition for identity fusion but are distinguished from the concept as they are not defined by the principles of (1) agentic-personal self, (2) identity synergy, (3) relational ties, and (4) irrevocability (Swann et al., 2012).

**FIGURE 1:**
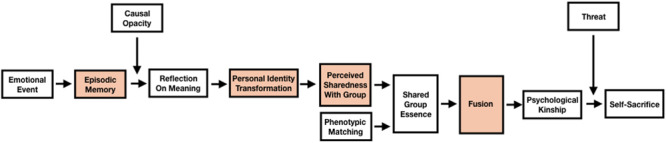
Shared pathway model adapted from Whitehouse (2018, p. 2) with relevant variables highlighted.

The full theoretical model is complex and involves a series of causal components (Figure 1). However, Whitehouse (2018, pp. 11–12) has urged researchers to view the model as presenting a discrete set of distinct but interlocking “testable hypotheses” that require targeted empirical validation. The goal of this paper then is to test one segment of the theorized relationships using unanalyzed variables from an existing dataset of responses collected from over a thousand Indonesian Muslims, including members of the general public and two Islamic organizations (Kavanagh et al., 2019b).

Specifically, we test the validity of the pathways proposed between memories of ‘group defining’ events (self-generated by the participants) and identity fusion with a relevant superordinate group (All Muslims). We do not measure shared essence directly as the dataset used in the study did not include a relevant measure. To do so we prompt individuals to recall a group and self-defining event related to their superordinate religious identity, in line with common methods used to make social identities salient (Andersen et al., 2007; McLeish and Oxoby, 2011; Ford et al., 2013). Then ask them to rate how far they view the event as being (1) self-transformative and (2) a shared experience with other group members. Finally, we use responses to these two measures to examine whether they are independently, or interactively, associated with levels of identity fusion with the superordinate group: All Muslims.

An Indonesian sample provides a relevant context in which to test the proposed model as, despite a longstanding reputation for religious syncretism and moderation, there are increasing concerns about growing support for extremist movements (Khisbiyah, 2009; Ward, 2009; Takwin et al., 2016; Arifianto, 2018). The sample selected not only includes the relevant measures but also should have a meaningful level of variability as the data was collected from a large, diverse array of Sunni Muslims in the Indonesian capital Jakarta. The original motivation for collecting the sample examined was to capture responses from members of a group that had ritual practices and organizational structures more conducive to producing fusion–the Prosperity and Justice Party (PKS)–and another more conducive to group identification–*Nahdlatul Ulama* (NU)–alongside a comparison group of unaffiliated members of the public. The groups were anticipated to represent different group bonding dynamics as, despite both being Sunni Islamic groups, the PKS and NU differ substantially in terms of ideology and regular practices.

*Nahdlatul Ulama* is the largest Islamic organization in Indonesia, noted for its moderate stance and endorsing the “middle path” (*wasathiyyah*) of Islam (Barton, 2014; Arifianto, 2016; Eliraz, 2016; Hosen, 2016). It also defines itself primarily as a religious social organization and officially does not participate directly in political contests^2^ (Barton, 2010, 2014; Sirry, 2010; Pribadi, 2013). Its practices revolve primarily around educational programs, such as administering of *Pesantren* (a traditional religious education system) and organizing collective sermons and large scale (non-compulsory) prayer sessions (Barton, 2010, 2014; Sirry, 2010; Pribadi, 2013). The PKS, in contrast, is an explicitly political and Islamist group with the stated aim of Islamizing Indonesia through cultural and political action (Nurdin, 2009; Machmudi, 2011). Membership within PKS is characterized by participation in intense religious study groups, where small groups (around 6–10 people) meet weekly to discuss Islamic doctrines and whether their actions have conformed to ideal Islamic practices (Machmudi, 2011). The PKS has well documented connections to transnational Islamic movements, most notably the Muslim Brotherhood in Egypt^3^ (Machmudi, 2011; Permata, 2016).

Returning to the ‘shared pathway’ model: the critical point is that high levels of identity fusion have been repeatedly demonstrated to have a strong predictive relationship with extreme pro-group action, including willingness to fight and die for the group (Swann et al., 2010a, b, 2014; Buhrmester et al., 2014; Swann and Buhrmester, 2015). Consequently, if we find support for the hypothesized pathways to fusion this could indicate that catalytic group-related experiences—that are regarded as transformative and shared—could constitute potential risk factors that increase the likelihood of an individual lending support, or engaging in, violent extremism when the contextual circumstances align, such as the presence of an ideology that endorses violence as legitimate.

### Previous Research

There have been a small number of previous studies that have examined the specific relationships explored in this paper. Whitehouse et al. (2017) using an online US Mechanical Turk (MTurk) sample, found a weak positive relationship (*r* = 0.24, *p* < 0.01) between ‘shared self-defining experiences’ and endorsement of self-sacrificial pro-group actions (Study 1) and, in a separate MTurk sample, found the relationship to be partially mediated by fusion (*b* = 0.40; Study 2). In the same paper, a study of 260 monozygotic and 246 dizygotic twins (Study 8) found that ‘shared experience’ was associated with identity fusion independent from genetic closeness, albeit the relationship was weak (*b* = 0.27). Another recent study (Newson et al., 2016) examined British Football fans and the connection between ‘self-shaping’ events and the degree to which individuals fuse with their club, where these events were significant wins and losses of their favored team. The results support a direct relationship between both positive and negative ‘self-shaping’ events and identity fusion levels (*r* = 0.40, *p* < 0.01). These results are encouraging but remain preliminary, and in all existing studies, measures of self-transformation and perceptions of shared experience have been aggregated, or the relationship has been simply inferred. As a result, there has not yet been a robust test of the causal chains hypothesized in the ‘shared pathways’ theoretical model (Figure 1).

Here, we seek to address this gap in the research literature by examining separate measures of (1) self-transformativeness and (2) the perception that memories are shared by other group members, and then testing the independent, and interactive, relationship of these variables and identity fusion with the group. A positive interactive effect is implied by the theoretical model but as noted above these variables have not been treated separately in previous research, so the current paper seeks to examine whether there is an interactive effect or if the measures and their effects should be treated independently in future research.

**FIGURE 2:**
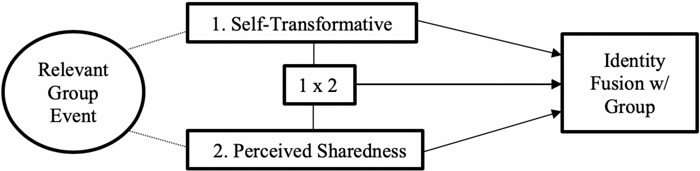
Conceptual model of hypothesized relationships.

A conceptual model of the relationships we will examine is provided below (Figure 2). To reduce researchers degrees of freedom (Kerr, 1998; Simmons et al., 2011; Wicherts et al., 2016) and increase transparency we preregistered our hypotheses and data analysis plans in line with the conceptual model outlined in Figure 2.

**FIGURE 3:**
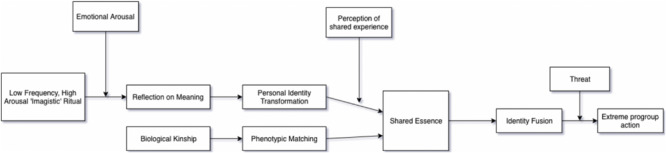
Theoretical model from Whitehouse and Kavanagh (in press).

We note here that in some formulations of Whitehouse’s ‘shared pathways’ theoretical model ‘perceptions of shared experience’ are positioned as a mediator acting between self-transformation and the sense of a ‘shared essence’ leading to identity fusion. We could therefore alternatively seek to test a mediation model (see Figure 3) but we opted not to do so for our main analysis, as we assessed the current evidence base as not yet strong enough to justify this more complex model. Instead, we examine the validity of the alternative model as an exploratory analysis.

### Main Hypotheses

Based on the ‘shared pathways’ theoretical model outlined in Whitehouse (2018), we predicted that in a large sample collected from Indonesian Muslims:

**H1a:** There will be a positive association between how self-transformative respondents’ rate ‘defining group events’ and their level of identity fusion with ‘All Muslims.’
**H1b:** There will be a positive association between respondents’ perception of having shared memories of ‘defining group events’ and their level of identity fusion with ‘All Muslims.’
**H1c:** That the main effects of both variables on identity fusion (with All Muslims) will be qualified by an interactive effect.
**H2:** That the pathways described in H1 will display a stronger association with identity fusion measures than with group identification with the same target group (with All Muslims).

### Alternative Hypothesis

Our justification for H2 is that there is a broad array of empirical results and theoretical discussion that suggests a stronger relationship between the perception of sharing a ‘group essence’ and identity fusion than with group identification (Swann et al., 2012; Whitehouse and Lanman, 2014; Whitehouse et al., 2017; Buhrmester et al., 2018; Whitehouse, 2018). The sense of possessing such a shared essence is theorized, in part, to emerge from an individual seeing relevant group events as (1) self-transformative and (2) believing their perception to be shared by other group members. However, Kavanagh et al. (2019b) found that, contrary to expectations, there was a stronger relationship between group identification and parochial progroup outcome measures than with fusion. An alternative hypothesis, therefore, based on the results reported in Kavanagh et al. (2019b) is that the relationship between self-transformative experiences and perceptions of shared memories will be stronger for group identification than identity fusion in this sample. We thus considered this as a possible alternative hypothesis but note that if observed whether it indicates a country-level effect, a pattern common to Muslim-majority countries, or a broader relationship counter to the existing theoretically literature will be impossible to determine without additional samples and further research.

## Materials and Methods

### Measures

We accessed an existing database of responses collected from Indonesian Muslims (see Kavanagh et al., 2019b) and extracted a number of variables. These included previously examined demographic control variables (age and gender), two measures of affiliation with religious group (fusion and identification), and potential confounding variables. The confounding variables selected were based on factors that were identified to be predictive of fusion and identification in Kavanagh et al. (2019b), specifically: group affiliation, intratextual fundamentalism, and level of religious practice. The two unanalyzed variables which are used to test the hypotheses for the present paper were self-reported ratings of (1) transformativeness and (2) perceived sharedness of a defining group experience. Further details of the relevant measures are detailed below.

#### Group-Defining Event Prompt

Participants were prompted to write about an event which they regarded as ‘defining’ for themselves and the fellow members of their group.^4^ The writing prompt was: *Describe in your own words an event related to Muslims in Indonesia/PKS/NU that you feel was most defining for how you view yourself and how other Muslims in Indonesia/PKS/NU members view themselves.* The intention here was to invoke a salient event that was regarded as personally transformative but, crucially, was group-relevant rather than being entirely personal. To make the intended contrast clearer, the next question alternatively asked the participants to list the three most memorable events in the history of their group (PKS/NU/Muslims in Indonesia) for group members. As relevant events were self-selected by participants and we desired variation in our key variables we kept all responses except for three in which the participants explicitly stated there was no defining event they could think of. There were 44 responses that did not provide accounts but answered follow up questions on their experience. We included these responses as we specified at the beginning of the questionnaire that participants could avoid answering questions if they felt uncomfortable or did not want to write down sensitive experiences. We did, however, check if excluding them altered observed patterns substantially but no differences were observed. For further details of the events mentioned see the results section.

#### Transformativeness

To assess transformativeness, we asked participants with reference to the group defining event they described, how far they agreed with three novel items, responding on a seven-point scale (1-strongly disagree to 7-strongly agree). The items were: ‘*My current self is the result of what Muslims experienced in Indonesia*’*;* ‘*What Muslims experienced in Indonesia, as I described, has a very significant role in shaping my current self*’*;* and ‘*If the events I described were not experienced by Muslims in Indonesia, I will probably be a totally different person today.*’ Responses were found to have good reliability, Cronbach’s α = 0.80, and a single mean-transformative score was computed, *M* = 4.6, *SD* = 1.5.

#### Perceived Sharedness

With reference to the group-defining event participants were asked to indicate how far they agreed with two items adapted from Whitehouse et al. (2017: Study 2), designed to measure the extent to which they viewed their memory of the event as shared with other members. A seven-point scale was used with the description at the lowest score indicating that ‘*My memories of what happened at that event are completely different from those of (my fellow group members)*’ and at the highest that ‘*My memories of what happened at that event are shared completely with (my fellow group members).*’ The second item similarly asked participants to indicate using the same seven-point scale whether ‘*their feelings about what happened at the event*’ were ‘completely different’ from their fellow group members (lowest score) or ‘shared completely’ (highest score). The scale had good reliability, Cronbach’s α = 0.82, and a single mean-sharedness score was computed, *M* = 4.6, *SD* = 1.5.

#### Group Affiliation

Group affiliation was determined via self-identification during data collection and individuals were categorized as belonging to PKS, NU, or as politically unaffiliated members of the public.

#### Intratextual Fundamentalism

Fundamentalism was measured using a three-item version of the intratextual fundamentalism scale (Hood et al., 2005), using a seven-point response scale (1-strongly disagree to 7-strongly agree) adapted to the Indonesian context in Muluk et al. (2013). Items included: ‘*Because the Qur’an can never be wrong, it must be understood literally according to what is written*’; and ‘*The Qur’an verses’ meaning are already clear, they mustn’t be debated.*’ Responses were found to have good reliability, Cronbach’s α = 0.79, and a single mean-fundamentalism score was computed, *M* = 5.6, *SD* = 1.5.

#### Religious Practice

A single score was calculated based on combined responses to four questions that asked about the frequency of four religious practices, responses were collected on a seven-point response scale (1 = never, 7 = always’). The practices referenced were: (1) *Practicing mandatory shalat (prayer) five times each day;* (2) *Fasting during Ramadan;* (3) *Practicing shalat sunnah (shalat that is encouraged but not mandatory)*; and (4) *attending religious gatherings, such as religious council, religious preaching, or religious discussion*. The scale items displayed moderate reliability (Cronbach’s α = 0.75, *M* = 5.8, *SD* = 1).

#### Identity Fusion

Identity fusion with the relevant categorical religious target group (‘All Muslims’) was measured using the pictorial identity fusion measure using a seven-point response scale (Swann et al., 2009). This item requests participants to select from seven images depicting two circles with varying degrees of proximity and overlap (one circle represents the participant and the other their group). The closer and greater the degree of overlap in the image, the higher the fusion score (1-no overlap, 7-complete overlap)^5^.

#### Group Identification

A single item measure of group identification was collected on a seven-point response scale (1–7) with higher scores indicating greater identification The wording of the measure was originally taken from the single item social identification measure (SISI; Postmes et al., 2013) but the specific wording was adapted during translation to: “*How strongly do you identify with ‘All Muslims”’* as this was found during translation processes to be easier to understand.

All items were translated from English to *Bahasa* Indonesian by a professional translator and then checked for consistency via back translation. Items with wording that were identified as hard to understand were discussed and edited to sound more natural at the expense of altering original wording (as per the SISI group identification measure).

### Preregistration and Data Archiving

The hypotheses, data analysis plan, and rationale for the study were all preregistered prior to examination of the data. The preregistration document is available from the Open Science Framework at the following address: https://osf.io/4jtkn/?view_only=0e4c439ced424007abcef16725c36b17^6^. The data used in all analyses reported in the manuscript is available to access on the Open Science Framework^7^.

### Ethics

All procedures for the study complied with the regulations of the School of Anthropology and Museum Ethnography Research Ethics Committee (Oxford University) and received approval (Ref No: SAME_C1A_16_015). Ethical clearance for the study in Indonesia was approved by the Ethics Committee Faculty of Psychology at the University of Indonesia (Ref No: 142/FPsi.Komite Etik/PDP.04.00/2017). The data collected from respondents was stored anonymously and all participants were provided with study information and required to complete a consent sheet.

### Sample

The dataset examined (Kavanagh et al., 2019b) included 1320 participants from three groups: 618 members of NU, 207 members of PKS, and 495 non-affiliated Muslims. Organizational identification was based on participants’ self-declaration in the surveys. Data collection targeting members of NU was conducted in the *Universitas Nahdhatul Ulama Indonesia* (UNUSIA) where most students are members of NU. Members of PKS were recruited through their weekly group meetings held in locations around Jakarta. General Muslims were recruited from non-affiliated members of the public located around university premises, and thus this group includes a significant portion of students. Participants were compensated for participation by payments of RP 50.000 (∼ $4). The mean age of the sample was 26.40 years (*SD* = 9.51), and was 53.6% male and 46.4% female. Ethnically, the majority of responses were Javanese (50.7%), followed by Sundanese (24.7%), Minangkabau (3.7%), Palembang (1.9%), Bornean (0.9%) and ‘other’ (18.2%). We included all participants who possessed no missing data for any of the variables analyzed and as a result omitted 69 participants, leaving *N* = 1248 participants. A G^∗^Power sensitivity analysis indicated that the sample afforded us 95% power to detect a small effect (*R*^2^ = 0.01) using multiple regression analysis (Faul et al., 2009).

### Pre-registered Analysis Plan

In order to test the three hypotheses associated with H1 we will execute a hierarchical regression analysis on the responses of our participants in which we predict identity fusion to All Muslims, using the following variables as predictors: (1) Self-transformation, (2) Perception of shared memories, (3) an interaction term of transformativeness and perceived sharedness. However, we will first run a baseline model which account for control variables: (1) group membership (dummy coded), (2) age, (3) sex, (4) Religious Practice Score, and (5) Intratextual Fundamentalism. Using this approach, we can determine to what extent the focal predictors improve the overall model beyond this baseline.

In order to test H2, we will compute the variance unique to both fusion and group identification; to do this we will predict the residuals for identity fusion (regressed on group identification) and group identification (regressed on identity fusion). These two new variables, which represent the unique variance of each construct (as well as measurement error) will then be the outcome of the model described in H1.

### Exploratory Analysis

We also plan, if the main relationships in H1 are observed, to conduct an exploratory analysis of a proposed mediation model in which ‘perception of shared experience’ mediates the relationship between ‘self-transformation’ and ‘identity fusion with All Muslims.’ We will assess the strength of the indirect and direct pathways and compare these with alternative models in which the order of the predictive relationships are reversed.

## Results

As anticipated, there was wide variation in the answers provided to the group-defining event prompt. Participants varied in how much depth they described events and valence was hard to distinguish since even when tragedies were referenced often the subsequent effects were noted as positive. One common example was references to a controversy involving the Christian governor of Jakarta who was accused and later sentenced for committing blasphemy against the Quran. Here, even participants who strongly disapproved of the governor’s statements frequently mentioned the feelings of unity that came from participating in the ‘212’ or ‘411’ protest demonstrations (names refer to the dates of the rallies).

The most commonly referenced topics were events related to: (1) religious practices, experiences or historical events connected with NU (37.3% with 5.6% focusing on the 1945 Jihad resolution), (2) the blasphemy controversy and subsequent protests (19.6%), (3) religious practices, experiences or historical events connected with PKS (11.8%, with 2.4% mentioning the arrest and detention of Luthfi Hasan Ishaaq PKS’ former leader), (4) Islamic holidays, regular religious practices, or Islamic teachings (14.9%), and (5) The events of the Gus Dur presidency (4%). Amongst the general public, the most common event mentioned was the blasphemy controversy which accounted for 42.6% of responses, followed by generic references to Islamic holidays, regular religious practices and teachings which accounted for 33.5% of responses.

**FIGURE 4:**
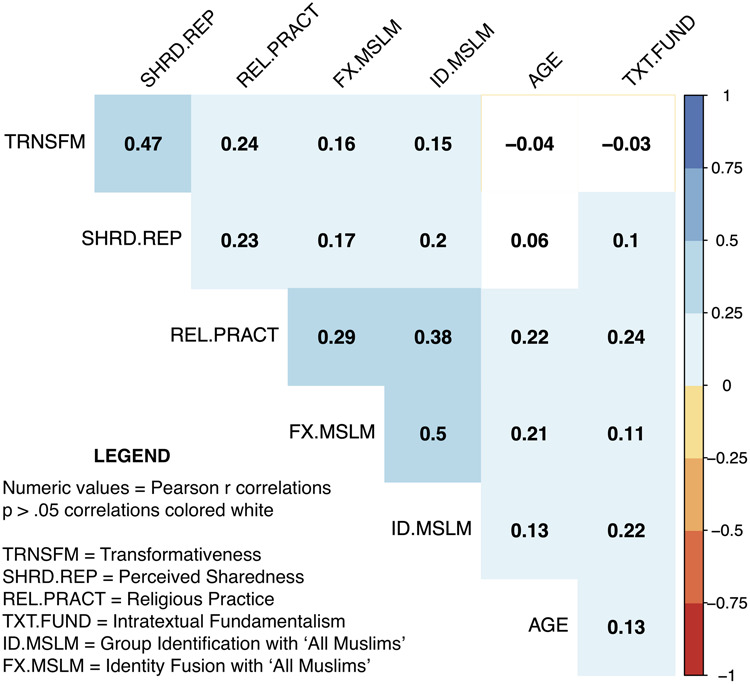
Correlation plot of study variables.

Figure 4 shows the correlations between all variables relevant to the present analyses. We note that transformativeness and perceived sharedness are moderately correlated (*r* = 0.47, *p* < 0.001), as are the measures of identity fusion and group identification (*r* = 0.50, *p* < 0.001).

### Hypothesis 1

**FIGURE 5:**
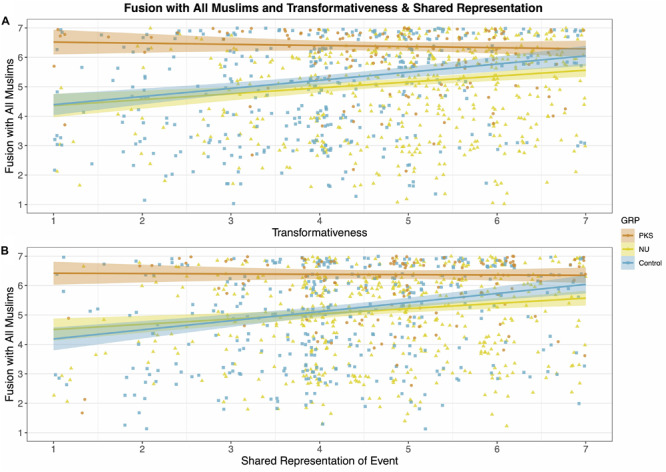
(A,B) Scatterplot of relationship between transformativeness, perceived sharedness and Fusion with ‘All Muslims.’ Jitter added to aid visibility.

First, we examined the simple correlations between the identity fusion measure and transformativeness (*r* = 0.16, *p* < 0.001) and perceived sharedness (*r* = 0.17, *p* < 0.001) which displayed weak but significant positive relationships. Figure 5 shows an illustrative scatterplot of the relationships broken down by groups, note that the PKS responses display a ceiling effect as most members selected the highest fusion response.

Following this, in adherence with our preregistered analysis plan, we conducted a hierarchical linear regression with identity fusion with ‘All Muslims’ as the outcome. The analyses were comprised of two models, in the first we included measures likely to confound the relationships of interest. Specifically, we included age, sex, group membership (dummy coded), and scores for intratextual fundamentalism and religious practice. The second model included all the measures from the first, but added standardized measures of transformativeness, perceived sharedness, and their interaction term. Table 1 shows the results of the regression analysis.

**TABLE 1:** Summary of regression models for H1.

	**Fusion to All Muslims**			
	**Model 1**		**Model 2**	
	***b***	**(*SE*)**	***b***	**(*SE*)**

Group (PKS)	0.67***	(0.14)	0.62***	(0.14)
Group (NU)	−0.23*	(0.11)	−0.31**	(0.11)
Sex (female)	−0.24**	(0.09)	−0.18*	(0.09)
Age	0.01**	(0.01)	0.02***	(0.01)
Religious practice	0.40***	(0.05)	0.35***	(0.05)
Textual fundamentalism	0.01	(0.03)	0.01	(0.03)
Transformativeness (1)	–	–	0.14***	(0.05)
Perceived sharedness (2)	–	–	0.10*	(0.05)
Interaction term (1) and (2)	–	–	−0.09*	(0.04)
Intercept	2.75***	(0.30)	3.10***	(0.31)
*R*^2^	0.14		0.16	
Adjusted *R*^2^	0.14		0.16	
Residual standard error	1.54		1.52	
*F* statistic	*F*(6,1208) = 33.45, *p* < 0.001		*F*(9,1205) = 26.10, *p* < 0.001	

*p < 0.05, **p < 0.01, ***p < 0.001, shaded variables were standardized.

Model 1 accounted for a modest amount of variation in the identity fusion outcome (*R*^2^_adj_ = 0.14), and the additions in model 2 provided a small, though significant, improvement (*R*^2^_adj_ = 0.16), *F*(3,1205) = 9.91, *p* < 0.001. The inclusion of the transformativeness and sharedness variables did not diminish the predictive value of the potentially confounding variables from model 1, which suggests that they accounted for their own unique variance. More specifically, both predictors demonstrated significant positive main effects in model 2, which accorded with H1a and H1b. However, the shared representation variable was on the edge of conventional significance values, *p* = 0.05, reducing confidence in the finding. The interaction term was also a significant predictor in the model, but the relationship was negative (counter to Hypothesis 1c).

### Hypothesis 2

In order to test our second hypothesis, that the pathways in H1 would display a stronger relationship with the fusion measure than with a matched group identification measure, we conducted another set of hierarchical linear regressions analyses. Given the moderate correlation observed between the fusion and identification measures, *r* = 0.50 (Figure 4) indicating shared variance of *R*^2^ = 0.25, we computed two new variables in order to make for a more meaningful test of our hypothesis. Specifically, we calculated the residual variance in the fusion measure, after running a regression with identification as the predictor variable and, vice versa, calculating the residual variance in identification after running a regression with fusion as the predictor variable. These values represent the unique variance associated with each construct, as well as an unknown amount of measurement error (this method was used in Kavanagh et al., 2019a). Using these residual values reduces the likely observable strength of relationships but provides greater resolution to detect unique relationships between transformativeness and perceived sharedness and the group identification and fusion outcomes. We hypothesized that, using these residual measures, identity fusion would still be predicted by the three factors listed in H1; and that there would be weaker relationships when the outcome was group identification.

We conducted a parallel analysis involving a two-stage linear regression with the newly calculated fusion and identification residual value. In the first stage, we entered potentially confounding demographic and religious variables, and in the second the three predictor variables of interest: transformativeness, perceptions of shared experience and their interaction term. Then we examined if there was any significant improvement between the model in stage 1 and stage 2 for both outcomes and explored the contributions made to the stage 2 models by the predictor variables.

First, we note the stage 2 model for both the fusion and group identification outcomes displayed significant model fit, and that the overall model fit was greatest for the group identification model. However, relative to the stage 1 model, a significant improvement with the addition of the target variables in stage 2 was only observed for the fusion residual, *F*(3,1205) = 6.82, *p* < 0.001, and not for the identification residual, *F*(3,1205) = 5.67, *p* = 0.10. This suggests that the focal predictors were more relevant predictors for the fusion residual, and that the identification residuals alternatively were more strongly associated with the confounding demographic and religious predictors entered in stage 1. The slightly better model fit for identification likely reflects the hypothesized relationship between ‘doctrinal’ religion, represented by ‘textual fundamentalism’ and ‘religious practices’ measures, and group identification processes (Whitehouse and Lanman, 2014).

**FIGURE 6:**
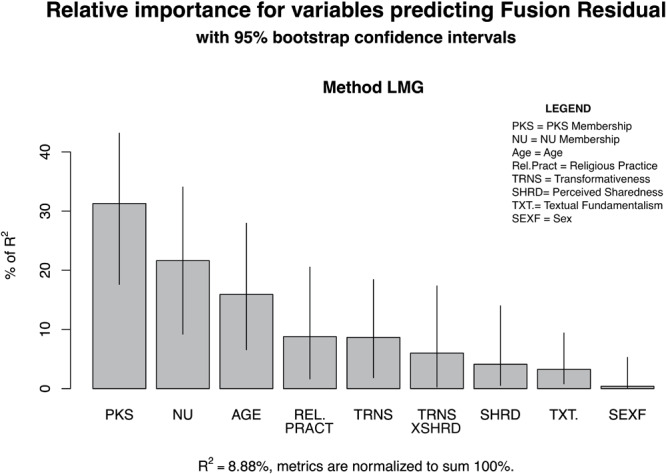
Barplot of relative contributions of variables to *R*^2^ predicting identification residual.

**FIGURE 7:**
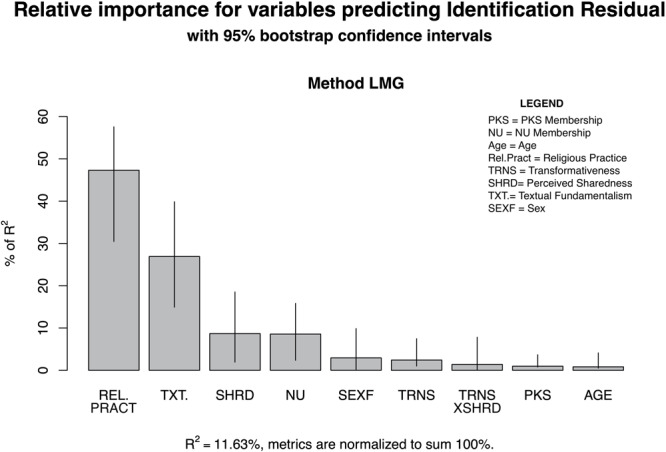
Barplot of relative contributions of variables to *R*^2^ predicting fusion residual.

Alternatively, we note that PKS membership was a strong predictor of higher fusion scores, while NU membership displayed a negative relationship. This was not the case with the group identification measure, supporting the contention that the constructs are distinguishable. In regard to the three target variables of interest, in the stage 2 model with the identity fusion residual as an outcome we found that only transformativeness displayed a significant main effect. There was no main effect observed for shared representation but as in H1 there was a weak negative relationship with the interaction. In contrast, in the stage 2 model with the identity fusion residual as an outcome shared representation was found to have a significant positive relationship (Table 2). To help visualize the difference we constructed plots, with error bars based on 500 bootstraps, that demonstrate the relative contribution of each variable to the overall variance explained in both models (Figures 6, 7).

**TABLE 2:** Summary of final regression models for H2.

	**Target group: All Muslims**			

**Outcome**	**Group identification**		**Identity fusion**	
	***b***	***SE***	***b***	***SE***
Group (PKS)	0.03	(0.09)	0.34***	(0.09)
Group (NU)	0.21	(0.07)	−0.26***	(0.07)
Sex (female)	−0.13*	(0.06)	–0.03	(0.06)
Age	0.00	(0.00)	0.01*	(0.00)
Religious practice	0.22***	(0.03)	0.08*	(0.03)
Textual fundamentalism	0.11***	(0.02)	−0.05**	(0.02)
Transformativeness (1)	–0.03	(0.03)	0.09**	(0.03)
Perceived sharedness (2)	0.07*	(0.03)	0.02	(0.03)
Interaction term (1) and (2)	0.03	(0.04)	−0.06**	(0.01)
Intercept	−1.93***	(0.19)	−0.26***	(0.19)
*R*^2^	0.12		0.09	
Adjusted *R*^2^	0.11		0.08	
Residual standard error	0.94		0.95	
*F* statistic	*F*(9,1205) = 17.63, *p* < 0.001		*F*(9,1205) = 13.05, *p* < 0.001	
Model improvement from S1	*F*(3,1205) = 5.67, *p* = 0.10, *R*^2^△ = 0.01		*F*(3,1205) = 6.82, *p* < 0.001, *R*^2^△ = 0.02	

* *p* < 0.05, ** *p* < 0.01, *** *p* < 0.001, shaded variables were standardized.

### Exploratory Analysis

**FIGURE 8:**
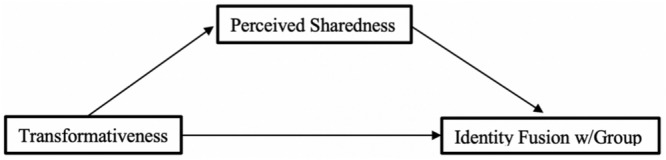
Proposed mediation model.

Bias corrected mediation analyses, based on 5,000 bootstrap samples, were conducted using PROCESS V3.0 (Model 4: Hayes, 2012) to examine a proposed mediation model wherein relationship between transformativeness and identity fusion with ‘All Muslims’ was mediated by perceived sharedness. A conceptual model of the proposed relationship is shown in Figure 8.

Analysis of the pathway outlined in Figure 8 did detect a partial mediation operating through perceived sharedness, *b* = 0.07, *SE* = 0.02, 95% CI [0.03, 0.10], although a direct effect remained, *b* = 0.11, *SE* = 0.03, 95% CI [0.04, 0.18]. However, robustness checks^8^ that examined alternative mediation pathways (exchanging sharedness and transformativeness and placing identity fusion in a moderating role) found partial mediations of similar magnitudes. It would thus be inappropriate to draw any strong conclusions about the directional relationships observed from these results.

## General Discussion

Using an existing dataset collected from three groups of Indonesian Muslims, we analyzed previously unexamined variables to test key relationships proposed by the ‘shared experiences pathway to fusion’ model (Whitehouse, 2018). In line with Open Science protocols (Nosek et al., 2018), our hypotheses and analyses were preregistered to restrict researchers degrees of freedom (Simmons et al., 2011) and reduce *post hoc* theorizing to fit the results observed (Kerr, 1998).

Our first hypothesis posited that after participants self-generated a memory of a group-defining event for Indonesian Muslims there would be positive associations between the degree to which the event was rated as self-transformative (H1a) and perceived as shared by the group (H1b) and identity fusion with the relevant categorical religious group (“All Muslims”). We also hypothesized that there would be an interactive positive relationship between these two variables and identity fusion with ‘All Muslims’ (H1c).

In support of our hypothesis we found that the ratings of self-transformativeness (*r* = 0.16, *p* < 0.001) and perceived sharedness (*r* = 0.17 *p* < 0.001) for self-generated defining group events were correlated with fusion to ‘All Muslims.’ Furthermore, a preregistered hierarchical regression predicting fusion with ‘All Muslims’ (after accounting for confounding demographic and religious variables) revealed significant positive independent associations for both transformativeness, (*b* = 0.14, *p* < 0.001) and perceived sharedness (*b* = 0.09, *p* = 0.05). The interaction of both variables also made a significant contribution to the model, although the relationship observed was unexpectedly negative (*b* = −0.09, *p* = 0.02), a point we will return to. We acknowledge, however, that caution is warranted as both the interaction term and the main effect of perceived sharedness are close to conventional thresholds of significance. Moreover, the total variance accounted for by their addition was small (*R*^2^ = 0.2). Nonetheless, our confidence in these results is increased as they were prespecified.

With those considerations in mind we interpret our findings as offering preliminary evidence for the association between identity fusion with a given group and the belief that group-defining events have been self-transformative. This is theoretically the result of such events becoming a core component of an individual’s personal identity and autobiography (Conway et al., 2004). However, we acknowledge that in the current dataset there was significant variation in the types of events being imagined and this is likely to have led to variation in how salient the events were for autobiographical identity.

A separate association was found with perceptions of group-defining experiences being shared with other group members, which has been theorized as providing fertile foundations for the development of identity fusion and psychological kinship (Swann et al., 2014; Whitehouse and Lanman, 2014). However, we note this relationship was not found in the regression models conducted to examine our second hypotheses that these relationships would be more associated with fusion over matched group identification measures. That the interaction between these two effects was negative was unexpected. A tentative explanation we considered was that if an event is regarded as self-transformative but also perceived to be widely shared then it may represent an event around which popular narratives, official accounts, or doctrinal descriptions exist and are disseminated. An illustration of this would be the research into memories of the 9/11 terrorist attack in America which Hirst et al. (2015) demonstrated were often strongly impacted by subsequent reporting.

The existence of an established narrative could diminish the event’s ability to serve as a catalyst for personal reflection and generate the kinds of relational bonds that are suggested as being fundamental to fusion processes (Gómez et al., 2011, pp. 918–919). The subversion of idiosyncratic reflection due to homogeneous accounts could instead lead to an alternative categorical form of bonding, such as group identification (Hogg, 2006; Hornsey, 2008). If the transformative nature of the event relies not on self-reflective *idiosyncratic* meaning-making but rather semantic knowledge of a shared cultural or historical narrative then it would seem to fall outside of the processes envisioned in the ‘shared experiences pathway to fusion’ model (Figure 1). Similarly, an event that is construed as a matter of doctrine would be more likely to generate group identification, according to Whitehouse and Lanman (2014).

Relatedly, the government and various religious groups in Indonesia, through their control of the education system, seek to instill shared historical narratives of the nation, Islamic history, and specific groups, including various transformative trials and challenges (Tan, 2012; Wieringa and Katjasungkana, 2018). Many of the events described by participants were of a public nature, and relevant to doctrine and shared history, and are topics of formal pedagogy. Previous findings on the same groups and participants as reported here show that group identification is more predictive than fusion for various progroup measures in Indonesia (Kavanagh et al., 2019b), and so it is notable that we still find evidence of the hypothesized relationships. However, we also caution that—counter to the account outlined here—we do not find any interactive effect of transformativeness and shared experiences in the regression models using group identification as an outcome.

To provide further detail, our second hypothesis was that the relationships between transformativeness and sharedness would be stronger for identity fusion with ‘All Muslims’ (Swann et al., 2009) than with a matched measure of group identification for the same target adapted from the single item social identity measure (Postmes et al., 2013). This prediction was based on the central position that processes of identity fusion occupy in the theoretical model (Figure 1) and on findings from the existing literature that distinguish group identification and fusion as related but distinct forms of group affiliation (Gómez et al., 2011; Swann et al., 2012; Whitehouse and Lanman, 2014; Bortolini et al., 2018). To test this prediction, we used the same predictive models as with hypothesis one, but this time the outcome variable was a residual of fusion after the shared variance with identification was removed (and vice versa).

In comparing the resulting models we found mixed support for the hypothesis: in the model with the fusion residual as an outcome, a significant main effect was only found for transformativeness (*b* = 0.09, *p* < 0.01) while their interaction term demonstrated a significant but weak negative relationship (*b* = −0.02, *p* < 0.01). There was no relationship observed with perceptions of shared experience. Alternatively, when the identification residual was the outcome, first we observed no significant improvement in the model when measures of transformativeness, shared representation, and their interaction were added as compared in stage 2. However, there was a relationship found with perceptions of shared experiences, *b* = 07, *p* = 0.02.

These results offer some support for the ‘shared experiences pathway to fusion’ model (2018), in that they support the importance of group-defining events being regarded as self-transformative and identify fusion but not for perceptions of shared experiences. We do not, however, find any strong support for the alternative hypothesis derived from the findings of Kavanagh et al. (2019b) that affiliation based on group identification is a more dominant process in Indonesia and thus that the variables expected to be associated with fusion might display a more robust relationship with identification. There was a relationship between identification and shared experiences, but the strength of the relationship was weak and fell close to conventional significance boundaries. This makes us unable to draw firm conclusions, but it is possible that the stronger relationship with identification reported in Kavanagh et al. (2019b) could be specific to progroup sacrifice measures. Moreover, given that all the relationships observed rely on self-reports it may be that fusion is a worse predictor of self-sacrificial progroup *sentiment* in Indonesia but still a better predictor of *behavior*. Further research in Indonesia, and other Muslim majority countries, will be necessary to determine the relevant predictive power of group identification and fusion for extreme progroup *behaviors*, such as violent protests or self-sacrifice.

We note that our findings, if valid, only demonstrate a relationship between transformative group-defining experiences and levels of fusion with a collective religious identity. Higher levels of fusion have been repeatedly found to be associated with greater endorsement of extreme progroup behavior, including violence against outgroup members (for an overview see Swann and Buhrmester, 2015). However, this is not an inevitable outcome of high levels in fusion, in the case where a group has strong prohibitions against violence and values that promote charitable self-sacrifice it may be that strong pro-group impulses can be channeled into socially beneficial behavior (Swann et al., 2012, p. 452).

Yet we note that almost one in five of our sample referenced the blasphemy controversy involving a Christian governor as their group-defining experience, and that amongst the sample from the general public this accounted for almost half of the responses. This suggests that there are widespread concerns about doctrinal conformity and potential outgroup threat, despite Islam being the overwhelming majority religion (followed by 87.2% of the population in the 2010 census). Relatedly, Whitehouse (2018, p. 2) identifies high levels of fusion combined with fears of outgroup threat as posing a potent foundation to motivate “extreme self-sacrifice for the group” as well as “less deadly forms of intergroup conflict… such as fan violence and hooliganism.” This is particularly concerning as in Indonesia hardline Islamist movements are growing in influence (Sakai and Fauzia, 2014; Muhtadi, 2018) and previous studies have revealed that organizations subscribing to extremist religious ideologies were the most likely to engage in lethal attacks (Asal and Rethemeyer, 2008; Webber et al., 2020). Further research is necessary to determine how contextual factors, including ideological commitment (Rogers et al., 2007; Putra and Sukabdi, 2014), interact with fusion though some research has found an interactive relationship with commitment to sacred values (Atran et al., 2014; Gómez et al., 2017).

Similarly, we recommend further research into the proposed mediating role of perceived sharedness between transformativeness and identity fusion. Our exploratory analysis of this proposed pathway did, in line with theoretical predictions, detect a partial mediation operating through perceived sharedness. However, the indirect pathway was weak and robustness checks found that relationships of similar sizes were observed for reversed causal models. Moreover, an important distinction between whether the perceptions of shared memories is generated by idiosyncratic self-reflective processes, or ascribed via more semantic or doctrinal processes, is not addressed by our current measures. In any case, we cannot draw strong inferences from the results observed and more targeted research is necessary in order to determine if the proposed mediating relationship exists or if the results observed in the current data, that suggest independent pathways and a negative interactive effect are replicated.

### Limitations

As this was a cross-sectional study, we are limited with regard to what causal inferences we can make. The theoretical model we tested in this paper presents a directional and causal chain of relationships, and the associations we found provide tentative support for these relationships. However, experimental and longitudinal data is more appropriate for assessing causal relationships. We would therefore encourage future research to further examine the causal direction of the relationships we report in the study.

We note also that using single item measures for our key outcomes is less than ideal and that the visual fusion scale represents a modification of an item that has been used elsewhere to measure group identification (Schubert and Otten, 2002). Nonetheless, we follow the existing literature in treating these as related but distinguishable group bonding constructs (Swann et al., 2009; Gómez et al., 2011; Swann and Buhrmester, 2015; Bortolini et al., 2018; Kavanagh et al., 2019a).

Indonesia is the most populace Muslim country in the world, the fourth most populace country overall, and the current study examines a large sample but there is a clear need for more comparable samples from Indonesia and other non-Western contexts. Currently, the majority of the fusion literature is based on research in Western, educated, industrialized, rich, and democratic (WEIRD) countries (Henrich et al., 2010; Swann and Buhrmester, 2015) and those that are from non-WEIRD contexts have tended not to feature or compare identification measures to the focal targets of identity fusion (Whitehouse et al., 2014; Gómez et al., 2017).

Although the relationships observed broadly accord with our pre-registered hypotheses it is important to recognize that the magnitude of the relationships are relatively weak, and that the overall variation accounted for in our regression models is between 8 and 16%. Moreover, the focal variables of interest only uniquely accounted for approximately 2% in models. We are cautious then not to not overstate the overall importance of the relationships reported. However, we believe it would be misguided to dismiss these findings on this basis of their effect size. The sample under investigation was diverse, as were the events described by the participants, and as such the relatively small amount of variance may be of less importance than whether the relationships detected prove to be valid. The best way to inform theory is with further analysis of large samples from diverse populations.

The diversity of events described and differences in how they were interpreted may have meant that the group-defining prompt served to activate different responses amongst participants. For instance, outgroup threat may have been induced by some descriptions and this could have resulted in identity affirmation processes (Sherman and Cohen, 2006, pp. 205–210) whereby people more strongly affirmed their identity as an Indonesian Muslim. However, we note that in context the prompt was affirming of the participants’ group membership as all participants were members of the relevant group mentioned. This is important as engaging in activities that remind people of group membership has also been found to reduce damaging implications for self-integrity from threatening events (Sherman and Cohen, 2006, p. 189). A deeper content analysis of the responses provided that examined, for instance, the proportion of references to self vs. group, level of affective engagement, or amount of reflection could be highly informative but is beyond the scope of this paper.

Another point to note is that we cannot tell from the current data whether the results observed are related to a short-term priming effect. Longitudinal studies would be required to address whether the relationships are observed in the absence of direct priming of group-defining experiences.

The current study presents a number of findings based on data collected from Indonesian Muslims: our sample included responses from a wide range of respondents, from members of a hardline Islamist group to ordinary non-affiliated members of the public. And while Indonesia is a country that is currently experiencing issues with religious extremism (Muluk et al., 2013; Burhani, 2014; Putra and Sukabdi, 2014; Arifianto, 2018; Putra et al., 2018), we want to emphasize that the purpose of the present article was not to address extremist behaviors or sentiment, which were not examined directly in this paper. Rather we sought to test specific components of a theoretical model that links self-defining events, identity fusion with a group, and *the potential* high levels of fusion have to foster extreme sacrifice (under specific conditions). We are, however, making no specific claims as to the nature and consequences of the fusion observed in this population. Nor are we claiming that the pathway under discussion is the *only* relevant pathway for predicting extreme behaviors, this is a complex topic and there are inevitably multiple pathways (Reicher et al., 2008; Gómez et al., 2017; Ginges, 2019). For more targeted discussion of what this specific sample reveals about links between fundamentalism, fusion, group identification, and parochial attitudes in Indonesia see both Kavanagh et al. (2019b) and Yustisia et al. (2020).

## Conclusion

We present here mixed support for the ‘shared experiences pathway to fusion’ model. There is substantial evidence highly fused individuals are more likely to endorse and engage in extreme pro-group actions (Swann et al., 2010a, b, Swann et al., 2014; Whitehouse et al., 2017; Bortolini et al., 2018; Kavanagh et al., 2019a). However, we note that fusion can serve as a means to motivate heroic self-sacrificing acts of devotion and kindness just as effectively as brutal suicide attacks. Identify fusion describes the nature of a specific relationship to a social group, and the strength of commitment to the values of that group but the nature of those values are group and context dependent (Swann et al., 2012). Here, we find that among a diverse group of Indonesian Muslims - who range from general members of the public to active members of hardline political Islamist groups - that there is a potential pathway to fusion with a categorical religious identity (‘All Muslims’) operating in parallel between the feeling that a defining group event is self-transformative and that the memory of the event is shared amongst group members. However, the relationships observed did not interact in a cumulative manner, instead a negative interaction was observed. Furthermore, the relationship with shared experiences and fusion was not replicated in analyses that sought to partial out the shared variance with a matched group identification measure. The self-transformative nature of group defining experiences may therefore be a stronger factor in contributing to the sense that an individual shares some ‘group essence’ with other relevant members. Further exploration of the causal pathways to identity fusion is required to establish if this relationship proves robust. This should be a priority for research on group cohesion given the established associations between identity fusion and extreme progroup sentiment and behavior.

Finally, in light of our acknowledged limitations, we make the following recommendations for future research. In order to better understand the *causal* relations in the model we encourage researchers to advance our own correlational work by using high-powered, longitudinal methodologies. We also encourage scholars interested in fusion to focus their attention on Indonesia, as Indonesia represents a nexus of multiple salient points of demography, psychology and religious fundamentalism, and there have been unexpected patterns observed with group identification acting as a stronger predictor of extreme progroup outcomes than fusion (Kavanagh et al., 2019b). More broadly we encourage a focus on collecting non-WEIRD samples in order to test theoretical generalizability especially where there are claims of universality. In line with this, scholars should be mindful of the utility and translatability of research measures that work well in WEIRD contexts. We are not suggesting such measures cannot work in non-WEIRD contexts, but simply that repeated independent validation is a necessary step in high-quality scholarship. This is why we recommend that scholars should compare and contrast the utility of competing constructs: fusion, in most cases, should be compared against group identification measures if a unique relationship is being posited. We have transparently presented our limitations, and hope to have set the stage for scholars to advance our own work.

## Data Availability Statement

All datasets generated for this study are included in the article/supplementary material. The preregistration document is available from the Open Science Framework (https://osf.io/gewmq/).

## Ethics Statement

The studies involving human participants were reviewed and approved by the School of Anthropology and Museum Ethnography Research Ethics Committee, The University of Oxford (Ref No: SAME_C1A_16_015), The Ethics Committee Faculty of Psychology, The University of Indonesia (Ref No: 142/FPsi.Komite Etik/PDP.04.00/2017). The patients/participants provided their written informed consent to participate in this study.

## Author Contributions

CK, RK, and HW prepared the preregistration. CK and RK conducted data analysis and prepared the figures. CK, RK, IP, and HW wrote the manuscript (listed in order of contribution).

## Conflict of Interest

The authors declare that the research was conducted in the absence of any commercial or financial relationships that could be construed as a potential conflict of interest.

## Footnotes

1 It should be noted that we are not suggesting individuals have a single group identity. All individuals possess multiple overlapping social/group identities (Roccas and Brewer, 2002). We are referring to the proposed ‘functional antagonism principle’ between social and personal identities (Spears, 2001).

2 Although most of the followers of NU are not directly involved in politics historically it has had greater involvement. Previously members within NU even created a political party under the name “Nahdlatul Ulama” and won seats in elections in 1950s. This group was eventually forced to merge into the United Development Party (PPP) with other Islam-based political parties in 1973 (Jones, 1984).

3 The PKS emerged from the religious movement *Jemaah Tarbiyah* during the 1980s–1990s and has been described as the “Indonesian branch of the Egyptian Muslim Brothers” (Permata, 2016, p. 29).

4 The relevant groups were fellow members of PKS or NU, or fellow Indonesian Muslims for the general public sample. Although these instructions meant that the groups referenced in the accounts varied, Kavanagh et al. (2019b) reported that for all three groups, aside from family, ‘All Muslims’ was the most salient group level identity on measures of fusion and identification.

5 Kavanagh et al. (2019b) collected measures of fusion and identity toward a number of other targets, including the categorical religious identity discussed here. In the present article, we are only concerned with this single target community.

6 Please note this is the blinded review link. If accepted this address will need to be amended.

7 https://osf.io/gewmq/

8 We note that there are criticisms of the practice of examining reversed causal models in mediation (Thoemmes, 2015) but that others recommend them as useful (Fiedler et al., 2018) to avoid drawing unwarranted causal conclusions.

## References

[B1] Andersen S. Moskowitz D. Blair I. V. Nosek B. (2007). “Automatic thought,” in *Social Psychology: Handbook of Basic Principals*, eds Kruglanski A. W. Higgins E. T. (New York, NY: Guilford Publications), 133–172.

[B2] Arifianto A. R. (2016). *Islam Nusantara: NU’s Bid to Promote “Moderate Indonesian Islam” Commentary No. 114; RSIS Commentaries.* Singapore: Nanyang Technological University, 1–3.

[B3] Arifianto A. R. (2018). Islamic campus preaching organizations in Indonesia: promoters of moderation or radicalism? *Asian Secur.* 15 323–342. 10.1080/14799855.2018.1461086

[B4] Asal V. Rethemeyer R. K. (2008). The nature of the beast: organizational structures and the lethality of terrorist attacks. *J. Polit.* 70 437–449. 10.1017/s0022381608080419

[B5] Atran S. Sheikh H. Gómez A. (2014). Devoted actors sacrifice for close comrades and sacred cause. *Proc. Natl. Acad. Sci. U.S.A.* 111 17702–17703. 10.1073/pnas.1420474111 25472844 PMC4273409

[B6] Barton G. (2010). Indonesia: legitimacy, secular democracy, and Islam. *Polit. Policy* 38 471–496. 10.1111/j.1747-1346.2010.00244.x

[B7] Barton G. (2014). The Gülen movement, muhammadiyah and nahdlatul ulama: progressive Islamic thought, religious philanthropy and civil society in Turkey and Indonesia. *Islam Christ. Muslim Relat.* 25 287–301. 10.1111/j.1398-9995.2009.02325.x 20146728

[B8] Bortolini T. Newson M. Natividade J. C. Vázquez A. Gómez A. (2018). Identity fusion predicts endorsement of pro-group behaviours targeting nationality, religion, or football in Brazilian samples. *Br. J. Soc. Psychol.* 57 346–366. 10.1111/bjso.12235 29322509

[B9] Buhrmester M. D. Fraser W. T. Lanman J. A. Whitehouse H. Swann W. B. (2014). When terror hits home: fused Americans saw Boston bombing victims as “family” and rushed to their aid. *Self Identity* 14 253–270. 10.1080/15298868.2014.992465

[B10] Buhrmester M. D. Newson M. Vázquez A. Hattori W. T. Whitehouse H. (2018). Winning at any cost: identity fusion, group essence, and maximizing ingroup advantage. *Self Identity* 17 500–516. 10.1080/15298868.2018.1452788

[B11] Burhani A. N. (2014). Treating minorities with fatwas: a study of the Ahmadiyya community in Indonesia. *Contemp. Islam* 8 285–301. 10.1007/s11562-013-0278-3

[B12] Cohen G. L. Garcia J. (2005). ‘ I am us’: negative stereotypes as collective threats. *J. Pers. Soc. Psychol.* 89 566–582. 10.1037/0022-3514.89.4.566 16287419

[B13] Conway M. A. Singer J. A. Tagini A. (2004). The self and autobiographical memory: correspondence and coherence. *Soc. Cogn.* 22 491–529. 10.1521/soco.22.5.491.50768

[B14] Dar-Nimrod I. Heine S. J. (2011). Genetic essentialism: on the deceptive determinism of DNA. *Psychol. Bull.* 137 800–818. 10.1037/a0021860 21142350 PMC3394457

[B15] Dugas M. Kruglanski A. W. (2014). The quest for significance model of radicalization: implications for the management of terrorist detainees. *Behav. Sci. Law* 32 423–439. 10.1002/bsl.2122 24802748

[B16] Eliraz G. (2016). *Indonesia’s Nahdlatul Ulama: A Tolerant, Inclusive Message to the Arab Middle East.* Washington, DC: Middle East Institute.

[B17] Faul F. Erdfelder E. Buchner A. Lang A.-G. (2009). Statistical power analyses using G^∗^ Power 3.1: tests for correlation and regression analyses. *Behav. Res. Methods* 41 1149–1160. 10.3758/brm.41.4.1149 19897823

[B18] Fiedler K. Harris C. Schott M. (2018). Unwarranted inferences from statistical mediation tests – An analysis of articles published in 2015. *J. Exp. Soc. Psychol.* 75, 95–102. 10.1016/j.jesp.2017.11.008

[B19] Ford J. O’Hare D. Henderson R. (2013). Putting the “we” into teamwork: effects of priming personal or social identity on flight attendants’ perceptions of teamwork and communication. *Hum. Fact.* 55 499–508. 10.1177/0018720812465311 23829025

[B20] Ginges J. (2019). The moral logic of political violence. *Trends Cogn. Sci.* 23 1–3. 10.1016/j.tics.2018.11.001 30497936

[B21] Gómez A. Brooks M. L. Buhrmester M. D. Vázquez A. Jetten J. Swann W. B. (2011). On the nature of identity fusion: insights into the construct and a new measure. *J. Pers. Soc. Psychol.* 100 918–933. 10.1037/a0022642 21355659

[B22] Gómez A. López-Rodríguez L. Sheikh H. Ginges J. Wilson L. Waziri H. (2017). The devoted actor’s will to fight and the spiritual dimension of human conflict. *Nat. Hum. Behav.* 1 673–679. 10.1038/s41562-017-0193-3 31024146

[B23] Haslam S. A. Oakes P. J. Turner J. C. McGarty C. (1996). “Social identity, self-categorization, and the perceived homogeneity of ingroups and outgroups: the interaction between social motivation and cognition,” in *Handbook of Motivation and Cognition. The Interpersonal Context*, Vol. 3 eds Sorrentino R. M. Higgins E. T. (New York, NY: Guildford Press), 182–222.

[B24] Hayes A. F. (2012). PROCESS: a versatile computational tool for observed variable mediation, moderation, and conditional process modeling. *White Paper* 1–39. Available online at: http://www.afhayes.com/public/process2012.pdf

[B25] Henrich J. Heine S. J. Norenzayan A. (2010). The weirdest people in the world? *Behav. Brain Sci.* 33 61–135. 20550733 10.1017/S0140525X0999152X

[B26] Hirst W. Phelps E. A. Meksin R. Vaidya C. J. Johnson M. K. Mitchell K. J. (2015). A ten-year follow-up of a study of memory for the attack of September 11, 2001: flashbulb memories and memories for flashbulb events. *J. Exp. Psychol. Gen.* 144 604–623. 10.1037/xge0000055 25751741

[B27] Hogg M. A. (2006). “Social identity theory,” in *Contemporary Social Psychological Theories*, ed. Burke P. J. (Palo Alto, CA: Stanford University Press), 111–136.

[B28] Hogg M. A. Turner J. C. (1987). Intergroup behaviour, self-stereotyping and the salience of social categories. *Br. J. Soc. Psychol.* 26 325–340. 10.1111/j.2044-8309.1987.tb00795.x

[B29] Hood R. W. Hill P. C. Williamson W. P. (2005). *The Psychology of Religious Fundamentalism.* New York, NY: Guilford Press.

[B30] Hornsey M. J. (2008). Social identity theory and self-categorization theory: a historical review. *Soc. Pers. Psychol. Compass* 2 204–222. 10.1037/bul0000156 29999335

[B31] Hosen N. (2016). *Islam Nusantara: A Local Islam with Global Ambitions?* Melbourne: Indonesia at Melbourne.

[B32] Jones S. (1984). The contraction and expansion of the” Umat” and the role of the nahdatul ulama in Indonesia. *Indonesia* 38 1–20.

[B33] Kavanagh C. M. Jong J. McKay R. Whitehouse H. (2019a). Positive experiences of high arousal martial arts rituals are linked to identity fusion and costly pro-group actions. *Eur. J. Soc. Psychol.* 49 461–481. 10.1002/ejsp.2514 31598015 PMC6774318

[B34] Kavanagh C. M. Wibisono S. Kapitány R. Yustisia W. Putra I. E. Rufaidah A. (2019b). Exploring the role of identity fusion and group identification in predicting parochialism amongst Indonesian Islamic groups [Preprint]. *PsyArXiv [Preprint]*

[B35] Kerr N. L. (1998). HARKing: hypothesizing after the results are known. *Pers. Soc. Psychol. Rev.* 2 196–217. 10.1207/s15327957pspr0203_4 15647155

[B36] Khisbiyah Y. (2009). “Contested discourses on violence, social justice, and peacebuilding among Indonesian Muslims,” in *Peace Psychology in Asia*, eds Noor N. Montiel C. (New York, NY: Springer), 123–145. 10.1007/978-1-4419-0143-9_7

[B37] Kruglanski A. W. Bélanger J. J. Gunaratna R. (2019). *The Three Pillars of Radicalization: Needs, Narratives, and Networks.* New York, NY: Oxford University Press.

[B38] Kruglanski A. W. Chen X. Dechesne M. Fishman S. Orehek E. (2009). Fully committed: suicide bombers’ motivation and the quest for personal significance. *Polit. Psychol.* 30 331–357. 10.1111/j.1467-9221.2009.00698.x

[B39] Kruglanski A. W. Gelfand M. J. Bélanger J. J. Sheveland A. Hetiarachchi M. Gunaratna R. (2014). The psychology of radicalization and deradicalization: how significance quest impacts violent extremism. *Polit. Psychol.* 35 69–93. 10.1111/pops.12163

[B40] Machmudi Y. (2011). *Islamizing Indonesia: The Rise of Jemaah Tarbiyah and the Prosperous Justice Party (PKS).* Canberra: Australian National University Press.

[B41] McLeish K. N. Oxoby R. J. (2011). Social interactions and the salience of social identity. *J. Econ. Psychol.* 32 172–178. 10.1016/j.joep.2010.11.003

[B42] Milla M. N. Putra I. E. Umam A. N. (2019). Stories from jihadists: significance, identity, and radicalization through the call for jihad. *Peace Confl.* 25 111–121. 10.1037/pac0000371

[B43] Muhtadi B. (2018). Explaining the 2016 islamist mobilisation in Indonesia: religious intolerance, militant groups and the politics of accommodation AU - Mietzner, Marcus. *Asian Stud. Rev.* 42 479–497. 10.1080/10357823.2018.1473335

[B44] Muluk H. Sumaktoyo N. G. Ruth D. M. (2013). Jihad as justification: national survey evidence of belief in violent jihad as a mediating factor for sacred violence among Muslims in Indonesia. *Asian J. Soc. Psychol.* 16 101–111. 10.1111/ajsp.12002

[B45] Newson M. Buhrmester M. D. Whitehouse H. (2016). Explaining lifelong loyalty: the role of identity fusion and self-shaping group events. *PLoS One* 11:e0160427. 10.1371/journal.pone.0160427 27508386 PMC4980014

[B46] Nosek B. A. Ebersole C. R. DeHaven A. C. Mellor D. T. (2018). The preregistration revolution. *Proc. Natl. Acad. Sci. U.S.A.* 115 2600–2606. 10.1073/pnas.1708274114 29531091 PMC5856500

[B47] Nurdin A. A. (2009). *Islamic Political Party and Democracy: A Comparative Study of PKS in Indonesia and PAS in Malaysia (1998-2005).* Singapore: National University of Singapore.

[B48] Permata A.-N. (2016). “A study of the internal dynamics of the prosperous justice party and jamaah tarbiyah,” in *Islam, Politics and Change: The Indonesian Experience After the Fall of Suharto*, eds van Dijk K. Zuhri S. (Leiden: Leiden University Press), 29–78.

[B49] Postmes T. Haslam S. A. Jans L. (2013). A single-item measure of social identification: reliability, validity, and utility. *Br. J. Soc. Psychol.* 52 597–617. 10.1111/bjso.12006 23121468

[B50] Pribadi Y. (2013). Religious networks in Madura: Pesantren, Nahdlatul Ulama, and kiai as the core of santri culture. *Al-Jami’ah J. Islam. Stud.* 51 1–32.

[B51] Putra I. E. Holtz P. Rufaedah A. (2018). Who is to blame, the victims or the perpetrators? A study to understand a series of violence targeting the accused heretic group Ahmadiyya. *Psychol. Relig. Spiritual.* 10 166–173. 10.1037/rel0000186

[B52] Putra I. E. Sukabdi Z. A. (2013). Basic concepts and reasons behind the emergence of religious terror activities in Indonesia: an inside view. *Asian J. Soc. Psychol.* 16 83–91. 10.1111/ajsp.12001

[B53] Putra I. E. Sukabdi Z. A. (2014). Can Islamic fundamentalism relate to nonviolent support? The role of certain conditions in moderating the effect of Islamic fundamentalism on supporting acts of terrorism. *Peace Confl.* 20 583–589. 10.1037/pac0000060

[B54] Reicher S. Haslam S. A. Rath R. (2008). Making a virtue of evil: a five-step social identity model of the development of collective hate. *Soc. Pers. Psychol. Compass* 2 1313–1344. 10.1111/j.1751-9004.2008.00113.x

[B55] Roccas S. Brewer M. B. (2002). Social identity complexity. *Pers. Soc. Psychol. Rev.* 6 88–106. 10.1207/s15327957pspr0602_01

[B56] Rogers M. B. Loewenthal K. M. Lewis C. A. Amlôt R. Cinnirella M. Ansari H. (2007). The role of religious fundamentalism in terrorist violence: a social psychological analysis. *Int. Rev. Psychiatry* 19 253–262. 10.1080/09540260701349399 17566903

[B57] Rosenberg M. (1987). “Depersonalisation: the loss of personal identity,” in *Self and Identity: Perspectives Across the Lifespan*, eds Honess T. Yardley K. M. (Abingdon: Routledge), 193–206.

[B58] Sakai M. Fauzia A. (2014). Islamic orientations in contemporary Indonesia: Islamism on the rise? *Asian Ethn.* 15 41–61. 10.1080/14631369.2013.784513

[B59] Schubert T. W. Otten S. (2002). Overlap of self, ingroup, and outgroup: pictorial measures of self-categorization. *Self Identity* 1 353–376. 10.1080/152988602760328012

[B60] Sherman D. K. Cohen G. L. (2006). The psychology of self-defense: self-affirmation theory. *Adv. Exp. Soc. Psychol.* 38 183–242. 10.1016/s0065-2601(06)38004-5

[B61] Sim J. J. Goyle A. McKedy W. Eidelman S. Correll J. (2014). How social identity shapes the working self-concept. *J. Exp. Soc. Psychol.* 55 271–277. 10.1016/j.jesp.2014.07.015

[B62] Simmons J. P. Nelson L. D. Simonsohn U. (2011). False-positive psychology: undisclosed flexibility in data collection and analysis allows presenting anything as significant. *Psychol. Sci.* 22 1359–1366. 10.1177/0956797611417632 22006061

[B63] Sirry M. (2010). The public expression of traditional Islam: the Pesantren and civil society in post-Suharto Indonesia. *Muslim World* 100 60–77. 10.1111/j.1478-1913.2009.01302.x

[B64] Spears R. (2001). “The interaction between the individual and the collective self: self-categorization in context,” in *Individual Self, Relational Self, Collective Self*, eds Sedikides C. Brewer M. B. (Hove: Psychology Press), 171–198.

[B65] Swann W. B. Buhrmester M. D. (2015). Identity fusion. *Curr. Dir. Psychol. Sci.* 24 52–57.

[B66] Swann W. B. Buhrmester M. D. Gómez A. Jetten J. Bastian B. Vázquez A. (2014). What makes a group worth dying for? Identity fusion fosters feelings of familial ties, promoting self-sacrifice. *J. Pers. Soc. Psychol.* 106 912–926. 10.1037/a0036089 24841096

[B67] Swann W. B. Gómez A. Dovidio J. F. Hart S. Jetten J. (2010a). Dying and killing for one’s group: identity fusion moderates responses to intergroup versions of the trolley problem. *Psychol. Sci.* 21 1176–1183. 10.1177/0956797610376656 20622141

[B68] Swann W. B. Gómez A. Huici C. Morales J. F. Hixon J. G. (2010b). Identity fusion and self-sacrifice: arousal as a catalyst of pro-group fighting, dying, and helping behavior. *J. Pers. Soc. Psychol.* 99 824–841. 10.1037/a0020014 20649370

[B69] Swann W. B. Gómez A. Seyle D. C. Morales J. F. Huici C. (2009). Identity fusion: the interplay of personal and social identities in extreme group behavior. *J. Pers. Soc. Psychol.* 96 995–1011. 10.1037/a0013668 19379032

[B70] Swann W. B. Jetten J. Gómez A. Whitehouse H. Bastian B. (2012). When group membership gets personal: a theory of identity fusion. *Psychol. Rev.* 119 441–456. 10.1037/a0028589 22642548

[B71] Syed M. McLean K. C. (2016). Understanding identity integration: theoretical, methodological, and applied issues. *J. Adolesc.* 47 109–118. 10.1016/j.adolescence.2015.09.005 26522883

[B72] Tajfel H. Turner J. C. (1985). “The social identity theory of intergroup behavior,” in *Psychology of Intergroup Relations*, 2nd Edn, Vol. 2 eds Worchel S. Austin W. G. (Wokingham: Nelson-Hall), 7–24.

[B73] Takwin B. Mudzakkir A. Salim H. Ahnaf M. I. Hamdi A. Z. (2016). *Studies on Tolerance and Radicalism in Indonesia: Lessons from 4 Areas (Tasikmalaya, Yogyakarta, Bojonegoro and Kupang).* Jakarta: The Wahid Institute.

[B74] Tan C. (2012). *Islamic Education and Indoctrination: The Case in Indonesia.* Abingdon: Routledge.

[B75] Thoemmes F. (2015). Reversing arrows in mediation models does not distinguish plausible models. *Basic Appl. Soc. Psychol.* 37, 226–234. 10.1080/01973533.2015.1049351

[B76] Turner J. C. (1987). “A self-categorization theory,” in *Rediscovering the Social Group: A Self-Categorization Theory*, eds Turner J. C. Hogg M. A. Oakes P. J. Reicher S. D. Wetherell M. S. (Oxford: Blackwell), 42–67.

[B77] Vázquez A. Gómez Á. Ordoñana J. R. Swann W. B. Whitehouse H. (2017). Sharing genes fosters identity fusion and altruism. *Self Identity* 16 684–702. 10.1080/15298868.2017.1296887

[B78] Ward K. (2009). Non-violent extremists? Hizbut Tahrir Indonesia. *Aust. J. Int. Aff.* 63 149–164. 10.1080/10357710902895103

[B79] Webber D. Kruglanski A. Molinario E. Jasko K. (2020). Ideologies that justify political violence. *Curr. Opin. Behav. Sci.* 34 107–111. 10.1016/j.cobeha.2020.01.004

[B80] Whitehouse H. (1992). Memorable religions: transmission, codification, and change in divergent melanesian contexts. *Man* 27 777–797. 12287974

[B81] Whitehouse H. (2013). “Religion, cohesion and hostility,” in *Religion, Intolerance and Conflict: A Scientific and Conceptual Investigation*, eds Clarke S. Powell R. Savulescu J. (Oxford: Oxford University Press).

[B82] Whitehouse H. (2018). Dying for the group: towards a general theory of extreme self-sacrifice. *Behav. Brain Sci.* 41 1–64. 10.1017/S0140525X18000249 29409552

[B83] Whitehouse H. Kavanagh C. M. (in press). “What is the role of ritual in binding communities together?,” in *Oxford Handbook for the Cognitive Science of Religion*, ed. Barrett J. (Oxford: Oxford University Press).

[B84] Whitehouse H. Lanman J. A. (2014). The ties that bind us: ritual, fusion and identification. *Curr. Anthropol.* 55 674–695. 10.1086/678698

[B85] Whitehouse H. Jong J. Buhrmester M. D. Gómez A. Bastian B. Kavanagh C. M. (2017). The evolution of extreme cooperation via shared dysphoric experiences. *Sci. Rep.* 7:44292. 10.1038/srep44292 28290499 PMC5349572

[B86] Whitehouse H. McQuinn B. Buhrmester M. D. Swann W. B. (2014). Brothers in arms: Libyan revolutionaries bond like family. *Proc. Natl. Acad. Sci. U.S.A.* 111 17783–17785. 10.1073/pnas.1416284111 25385591 PMC4273349

[B87] Wicherts J. M. Veldkamp C. L. Augusteijn H. E. Bakker M. Van Aert R. Van Assen M. A. (2016). Degrees of freedom in planning, running, analyzing, and reporting psychological studies: a checklist to avoid p-hacking. *Front. Psychol.* 7:1832. 10.3389/fpsyg.2016.01832 27933012 PMC5122713

[B88] Wieringa S. E. Katjasungkana N. (2018). *Propaganda and the Genocide in Indonesia: Imagined Evil.* Abingdon: Routledge.

[B89] Yustisia W. Putra I. E. Kavanagh C. Whitehouse H. Rufaidah A. (2020). The role of religious fundamentalism and tightness-looseness in promoting collective narcissism and extreme group behavior. *Psychol. Relig. Spiritual.* 12, 231–240. 10.1037/rel0000269

